# Microcystins and Cyanobacterial Contaminants in the French Small-Scale Productions of Spirulina (*Limnospira* sp.)

**DOI:** 10.3390/toxins15060354

**Published:** 2023-05-24

**Authors:** Pierre-Etienne Pinchart, Amandine Leruste, Vanina Pasqualini, Felice Mastroleo

**Affiliations:** 1 UMR 6134 SPE, Université de Corse Pasquale Paoli (UCPP), 20250 Corte, France; pierre_etienne_pinchart@yahoo.fr (P.-E.P.); pasqualini_v@univ-corse.fr (V.P.); 2 Fédération des Spiruliniers de France (FSF), 34800 Clermont-l’Hérault, France; amandine@spiruliniersdefrance.fr; 3 Microbiology Unit, Nuclear Medical Applications, Belgian Nuclear Research Centre, SCK CEN, 2400 Mol, Belgium

**Keywords:** cyanotoxins, *Cyanobium*, *Jaaginema*, *Planktolyngbya*, *Leptolyngbya*, *Pseudanabaena*, *Cyanodictyon*, *Gomontiella*, *Phormidium*, BGAS

## Abstract

Spirulina is consumed worldwide, in the form of food or dietary supplements, for its nutritional value and health potential. However, these products may contain cyanotoxins, including hepatotoxic microcystins (MCs), produced by cyanobacterial contaminants. The French spirulina market has the particularity of being supplied half-locally by approximately 180 small-scale spirulina production farms. Data about this particular production and possible contaminations with other cyanobacteria and MCs are scarce. Thus, we collected the results of MC analyses and total cyanobacteria counts, carried out between 2013 and 2021, from 95 French spirulina producers who agreed to share their data. These data consisted of MC concentrations determined with an enzyme-linked immunosorbent assay (ELISA) using 623 dry spirulina samples and 105 samples of spirulina cultures. In addition, potentially unsafe samples of dry spirulina were further investigated through mass spectrometry, as duplicate analysis. We confirmed that the situation of the French spirulina production stayed within the safe regulatory level in terms of MC levels. On the other hand, the inventory of cyanobacterial contaminants, based on 539 count results, included 14 taxa. We present their prevalence, interannual evolution and geographical distribution. We also suggested improvements in cultivation practices to limit their propagation.

## 1. Introduction

Spirulina is the traditional and commercial name of edible filamentous cyanobacteria, which have first been assigned to the genera *Spirulina* and *Arthrospira* [1], and more recently to the genus *Limnospira* [2]. In this paper, the name ‘spirulina’ will refer to *Limnospira* (previously known as *Arthrospira*) spp. Spirulina is naturally present in alkaline fresh water, rich in carbonates, bicarbonates, nitrates, phosphates and iron. It grows mainly in the intertropical regions—Rift Valley lakes (Kenia, Mozambique), lake Chad, lake Paracas (Peru), lake Texcoco (Mexico), lake Lonar (India) [3]—and is also found in Serbia [4] and Siberia [5]. Spirulina, harvested in lake Texcoco was consumed by the Aztecs at the time of the Spanish conquest (16th century). The traditional consumption of spirulina harvested in lake Chad by the Kanembou led to the discovery of the nutritional richness of this cyanobacterium in the 1960s [6]. The exceptional nutritive value of spirulina has led the United Nations Food and Agriculture Organization (FAO) to consider spirulina a food of major interest against malnutrition for the food security and the response to emergency food situations [7]. Spirulina contains a set of specific molecules—c-phycocyanin, complex polysaccharides such as calcium spirulan and γ-linolenic acid—which have strong prophylactic and therapeutic potential, especially in the fields of cardiovascular diseases, viral infections, cancer prevention and therapy, immune response, as well as diabetes and cholesterol control [8,9].

Many cyanobacteria are known to produce various cyanotoxins, and blue-green algae dietary supplements (BGASs) have long been suspected of containing toxic cyanobacteria and cyanotoxins [10]. The latter can be classified according to their toxic effect. Among the hepatotoxins, microcystins (MCs) are hepatotoxic and cyclic heptapeptides, of which there are more than 270 congeners which are differentiated by amino acid variations in two positions and the removal or addition of methyl groups [11]. The MC-LR congener for instance, produced by *Microcystis aeruginosa* particularly, is the one that has been the most studied. It is considered to be among the most toxic forms and it is the subject of health recommendations [12]. The amino acid ADDA, specific to MCs and nodularins, is used in ELISA enzymatic analysis methods to detect MCs without the distinction of congeners. Analyses combining chromatographic and mass spectrometry methods (e.g., LC-MS/MS) are considered to be more precise and sensitive, but are limited to congeners (e.g., MC-LR) for which reference standards are available [13].

In 1996, the Upper Klamath Lake (Oregon), where the cyanobacteria *Aphanizomenon flos-aquae* is harvested for producing food supplement, experienced an extensive bloom of *Microcystis aeruginosa*, possibly producer of the hepatotoxic MCs. This event triggered a study to test the presence of MCs in BGAS and establish a regulatory limit. The presence of MCs in food supplements based on spirulina, marketed in the USA, was demonstrated in 1998, and a safe level for microcystins in BGAS was determined to be 1 µg/g (1 ppm) by the Oregon Department of Agriculture ODA in 1997 [14]. This regulatory level was established on the basis of the recommendation of tolerable daily intakes for chronic exposure to microcystin-LR, 0.04 µg MC-LR/(kg.day), established in 1997 by the WHO and confirmed in 2020 [12]. This regulatory standard level is also used as a reference in other countries [15]. Since then, the detections of MCs in spirulina samples from different markets (Australia, Belgium, China, Germany, Italy, India, Spain, Switzerland, USA) have shown highly variable results, ranging from non-detection to alarming values [14,16,17,18,19,20,21,22,23,24,25,26].

Potential toxin-producing cyanobacteria identified to date include species belonging to the following genera: *Microcystis, Planktothrix*, *Dolichospermum* (*Anabaena*), *Anabaenopsis*, *Fischerella*, *Geitlerinema*, *Leptolyngbya*, *Merismopedia*, *Phormidium, Nostoc* and *Synechocystis*—and others species: *Annamia toxica*, *Aphanocapsa cumulus*, *Calothrix parietina*, *Hapalosiphon hibernicus*, *Radiocystis fernandoi*, *Cyanobium bacillare*, *Arthrospira fusiformis*, *Limnothrix redekei* and *Trichormus variabilis* [27,28,29,30,31,32]. Some of these possibly toxin-producing cyanobacteria (*Calothrix*, *Phormidium*, *Microcystis*, *Nostoc*, *Anabaenopsis*, *Geitlerinema*, *Leptolyngbya*) have been identified through molecular analysis of dry spirulina samples [33]. Therefore, beside *Limnospira* sp., the presence of toxinogenic cyanobacterial contaminants is the most likely hypothesis to explain the presence of MCs in spirulina products [17,29,34]. Previous toxicity analysis proved that *Limnospira* spp. is safe to consume [35,36]. However, a final consensus on the toxinogenic nature of *Limnospira fusiformis* is not yet established, since two controversial studies have shown the presence of MCs produced by strains of *L. fusiformis* from Kenyan lakes [37,38].

Spirulina is produced in 22 countries around the world, for a total production of 56 kt (tons fresh weight) in 2019 [39]. It is used for human food, animal feed, cosmetics and pharmaceuticals. Industrial producers, mainly located in China, India and the USA, cultivate spirulina in open ponds, reaching 120 ha [8,40]. In France, 201 t of dried spirulina, i.e., 3/4 of European production, were produced in 2019 by 161 aquaculture farms [41,42] which are mostly members of the professional organization “Fédération des Spiruliniers de France” (FSF). This production, intended for human consumption, is based on small-scale spirulina culture methods inspired by the work of Ripley Fox and Jean-Paul Jourdan [43,44]. FSF members produce their spirulina in basins with an average area of 600 m^2^, protected by greenhouses. Production and harvesting are seasonal activities that generally take place from the March–April period to the September–October period. The biomass harvested by filtration is pressed to eliminate the residual culture medium before drying by air convection at a low temperature (maximum 50 °C).

The FSF and its affiliated producers have been engaged since 2013 in monitoring the presence of MCs and cyanobacterial contaminants in dry spirulina products and spirulina cultures. The data collected by the FSF are presented and examined in this paper in order to assess the microcystin risk of this small-scale production and propose control and risk management measures.

## 2. Results

### 2.1. Microcystins in Spirulina Products and Cultures

**Figure 1:**
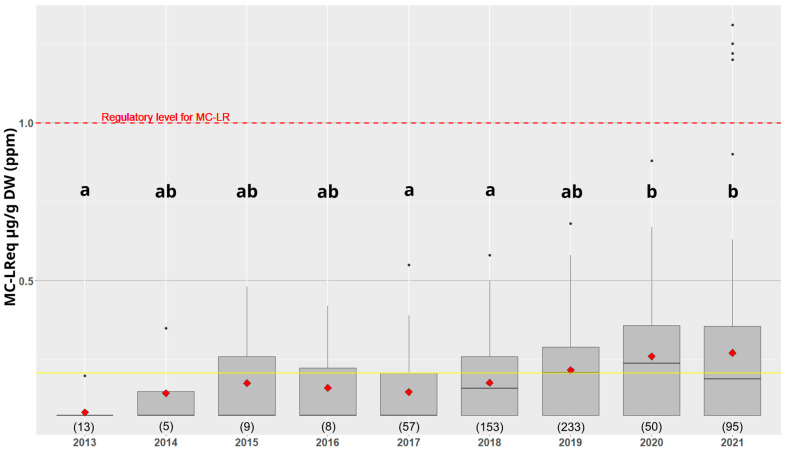
Microcystin-LR equivalent (MC-LReq) in dry spirulina products detected with ELISA assays between 2013 and 2021. The boxplot represents the dispersion of the MC level, the yellow line represents the mean over the years and the red diamonds represent the annual averages. The number of samples per year is indicated between the brackets. The letters a, b and ab group together the data series of which the means are not significantly different (Tukey’s test).

Between 2013 and 2021, 42% of the dry spirulina analysis results with the ELISA method were below the quantification limit—0.15 ppm MC-LR equivalent dry weight (MC-LReq DW)—and more than 99% of the results were below the regulatory level (1 ppm MC-LReq DW) (Figure 1). The average content of MCs in dry spirulina over the entire period studied is 0.21 ppm MC-LReq DW.

There are few results available for the years 2013 to 2016. From 2017, the data are more numerous and therefore more representative. There is an increase in the average levels of MCs between the years 2017 and 2018 (group a), and 2020 and 2021 (group b). The year 2019 is the most documented period in our study.

In 2021, four ELISA assay results were above the regulatory level (1 ppm MC-LReq DW). These four batches of spirulina, complemented by a fifth one below the regulatory level, were then analyzed using UHPLC-MS/MS to confirm these high levels of MCs before considering destroying the related product batches. The values found via UHPLC-MS/MS were lower than those obtained with ELISA (Table 1).

**Figure 2:**
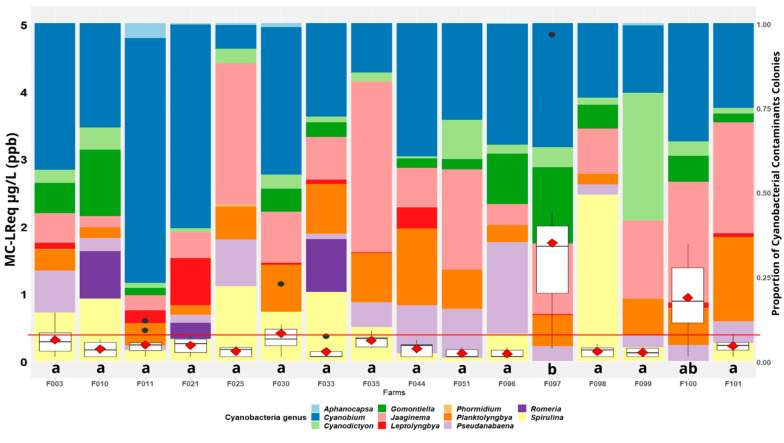
Cyanobacterial contaminants proportion and MC-LReq in spirulina culture samples (*n* = 105, year 2017) detected with ELISA assays. The boxplot represents the dispersion of the MC level and the red diamond represents the average for each farm. The red line represents the overall average (0.39 ppm MC-LReq). The letters a, b and ab group together the MC data series of which the means are not significantly different (Tukey’s test). The colored bands in the background represent the colonies, in proportion to the different cyanobacterial contaminant taxa encountered on each farm.

In 2017 specifically, we had access to both analyzes of MCs, using ELISA, and the total cyanobacteria counts on spirulina culture samples. The related levels of MC measured were relatively low, with an overall mean of 0.39 ppb (Figure 2), which is under the 1 ppb WHO guideline value for MC-LR in potable water, for instance [12]. Notably, 29% of the results of MC analysis in the cultures was below the limit of quantification (0.15 ppb).

The culture samples from two farms (F097 and F100) show the MC values significantly higher than those found in the other farms. The highest value encountered is 4.85 ppm (F097). Examination of the proportions of cyanobacterial contaminants, compared with the other farms, does not explain the high levels of MCs encountered in the samples from these two farms.

**Figure 3:**
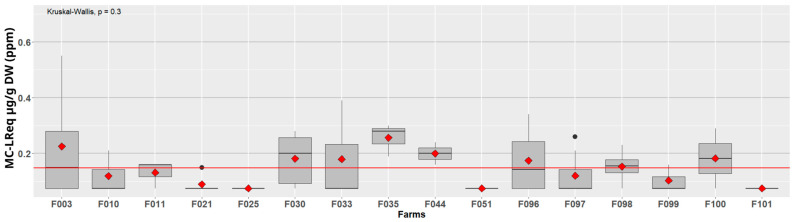
MC-LReq in dry spirulina products (57 samples, year 2017) detected with ELISA assays. The boxplot represents the dispersion of the MC level and the red diamond represents the average for each farm. The red line represents the overall average (0.15 ppm). The averages per farm are not significantly different according to Kruskal–Wallis test.

The levels of MCs found in the spirulina culture samples in 2017 (Figure 2) do not reflect the levels found in the related harvested dry products (Figure 3).

We retrieved twelve studies looking for MCs in spirulina products published to date (Table 2). Four of them used enzymatic methods (including three using ELISA) and nine used methods combining chromatography and mass spectrometry. The results of our 623 analyses of MCs using ELISA on dry spirulina showed a detection rate of 58% and a maximum value of 1.31 ppm, while the published results show a detection rate of 78% (18 positive samples out of 23) and a maximum value of 2.12 ppm. Studies using chemical analysis methods produced results which are also comparable in magnitude with our results obtained using UHPLC-MS/MS, with detection rates and levels of MCs lower than those encountered using the ELISA method.

### 2.2. Cyanobacterial Contaminants

**Figure 4:**
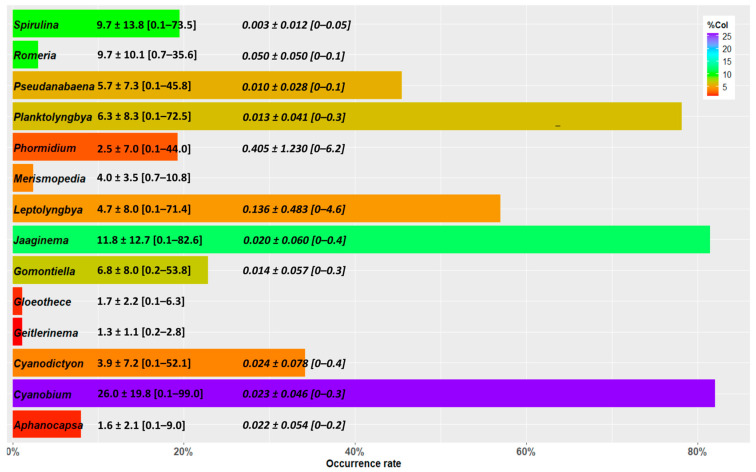
Cyanobacterial contaminants genus in spirulina cultures and dry products samples from 2017 and 2019 to 2021 (*n* = 539 samples). The length of the bars represents the occurrences of observations of each genus. Their color represents the average percentage of colonies (%Col) observed. The numbers following the genus names are relative to the percentages of the colonies observed—the mean ± SD [min.–max. values]—and, in italics, the percentages of biovolumes when available.

Fourteen genera of cyanobacteria (other than *Limnospira*) have been identified through microscopic observations in spirulina culture and dry product samples (Figure 4).

The two genera of cyanobacterial contaminants most frequently observed (*Cyanobium* and *Jaaginema*) belong to the Synechoccocales, which is the most represented order (5 genera out of 14). The next three most-represented genera (*Planktolyngbya*, *Leptolyngbya* and *Pseudanabaena*) belong to the Pseudanabaenales.

The numbers of colonies observed, expressed as a percentage of the total cyanobacteria (including *Limnospira*) in the samples are highly variable. According to this criterion, *Cyanobium* and *Jaaginema* are also the first two cyanobacterial contaminants.

The proportions of biovolumes of cyanobacterial contaminants are low, of the order of a thousandth, particularly due to the large biovolumes, generally greater than 99%, of the edible *Limnospira*. *Phormidium* is the contaminant which presents the largest biovolumes both on average and in maximum values.

**Figure 5:**
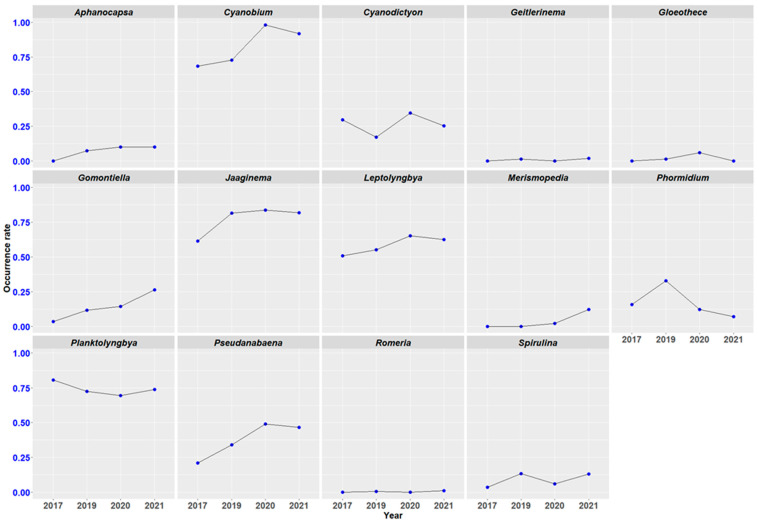
Interannual evolution of the occurrence rate of cyanobacterial contaminants in dry spirulina. Number of samples per year: 2017 (*n* = 57), 2019 (*n* = 232), 2020 (*n* = 49), 2021 (*n* = 99). The 75 farms located in our dataset are spread over the entire territory of mainland France, with a predominance in the South, Southeast and West regions. No geographical trend is observed for the taxonomic diversity of cyanobacterial contaminants which ranges from 3 to 11 taxa (genus) per farm, with an average between 6 and 7 genera. The cyanobacteria most often identified in our samples have a wide geographical distribution (Figure 6).

The interannual evolution of the frequency, or occurrence rate, of cyanobacterial contaminants in dry spirulina (flakes) is relatively stable (Figure 5). The most frequently observed taxa, with an occurrence rate greater than 50%, remain dominant from year to year. In this group, *Cyanobium* almost reached 100% occurrence in 2020. Conversely, the less often observed taxa, *Gloeothece* and *Romeria*, are not observed every year. There is an increase in the observations of *Gomontiella* and *Pseudanabaena.*

**Figure 6:**
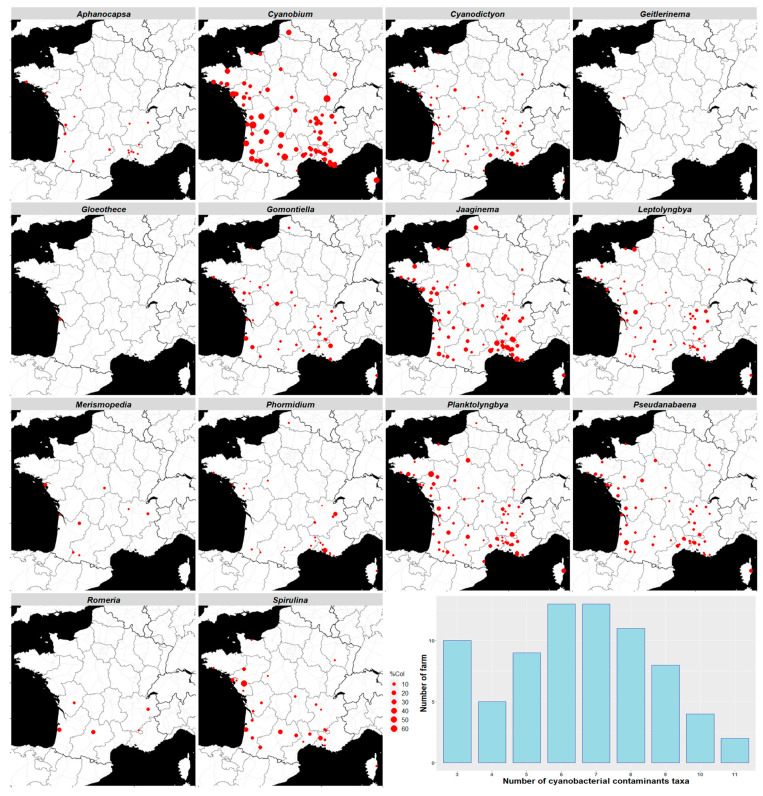
Geographical distribution of cyanobacterial contaminants. The red dots indicate the farm location where the cyanobacteria have been identified. The size of the dots is proportional to the average percentage of the colonies of the different cyanobacteria in each farm. *n* = 75 farms. The histogram represents the distribution of the numbers of cyanobacterial contaminant taxa per farm (mean = 6.48).

*Cyanobium*, *Jaaginema*, *Planktolyngbya*, *Pseudanabaena* and *Leptolyngbya* are present in more than 70% of the 75 located farms. Overall, the intermediate taxa (20 to 50% occurrence rate), *Cyanodicton*, *Gomontiella*, *Spirulina* and *Phormidium*, have geographical extents in proportion to their occurrence rate, while several taxa are closely related to specific zones, such as *Romeria*, *Gloeothece* and *Geitlerinema* (Figure 6).

**Figure 7:**
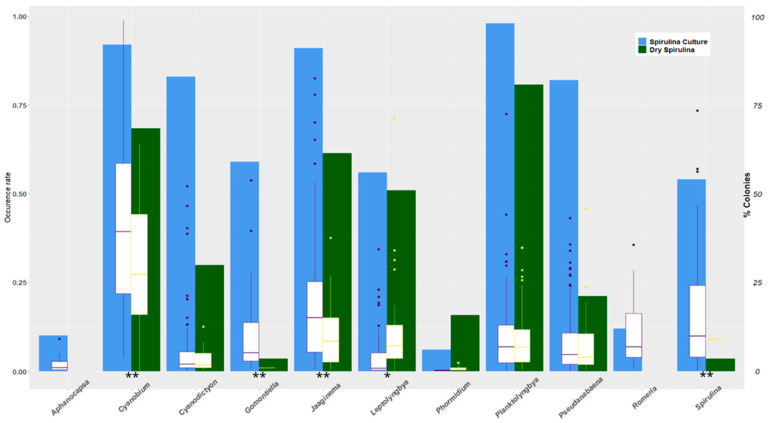
Evolution of contaminants from cultivation to harvested product. The blue and green bars represent the occurrence rates of the contaminants, respectively, in the spirulina culture samples and the dry harvested products. The boxplots represent the dispersion of the colonies, expressed as a percentage of the total cyanobacteria including *Limnospira*, of the different kinds of contaminants detected in 2017 in 16 farms, respectively, in the spirulina culture samples (in purple, *n* = 100) and the dry harvested products (in yellow, *n* = 57). The taxa with significantly different relative abundances between cultures and dry products are indicated by stars (*: *p* < 0.05; **: *p* < 0.01).

The cyanobacteria count of spirulina culture samples and spirulina dry products in 2017 enables comparing the presence of cyanobacterial contaminants before and after harvest. Except for *Phormidium*, the rate of occurrence of cyanobacterial contaminants is higher in spirulina culture samples than in harvested dry products (Figure 7).

**Figure 8:**
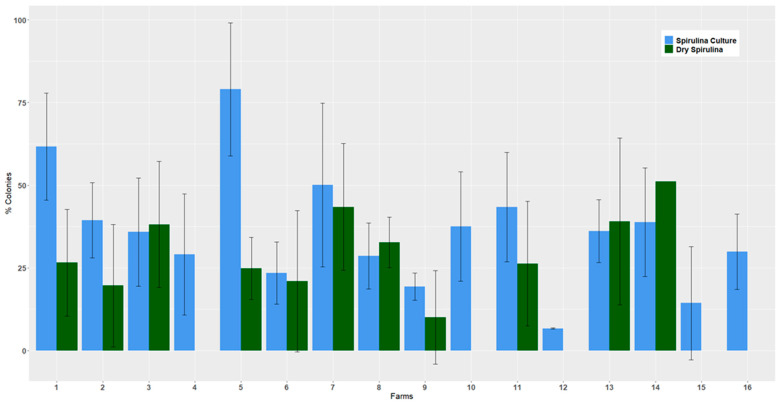
Evolution of *Cyanobium* from cultivation to harvested product. The bars represent the means of the relative abundances of *Cyanobium* colonies, expressed as a percentage of total cyanobacteria, respectively, in the samples of spirulina cultures (*n* = 100) and the products harvested (dry spirulina, *n* = 57) for 16 farms.

A great variability is observed in the percentages of colonies observed for each taxon, both for the spirulina culture samples and the harvested dry products. However, we can observe trends in the evolution of contamination which ranged, depending on the taxa, from the disappearance to an increase in colonies between crops and harvested products. Among the picocyanobacteria, *Aphanocapsa* and *Romeria* are present in crops and absent from harvested products, while *Cyanobium* is significantly decreasing. For the later taxon, which is present in 92% of the samples of spirulina cultures and identified in all the farms that participated in the culture analyses, we have enough data to make a comparison, farm by farm, of the evolution of the colonies between the spirulina culture samples and the products harvested. This comparison highlights the differences in the evolution of the number of *Cyanobium* colonies, from disappearance to increase, depending on the farm (Figure 8).

## 3. Discussion

None of the 623 food supplement samples obtained from French small-scale spirulina producers exceeded the regulatory level (1 ppm) of MCs. The average value was 0.21 ppm, taking into account a value of 0.075 ppm for the 42% of results below the LQ (0.15 ppm). Based on these results, we can then estimate that the daily consumption of 3 g of spirulina (doses generally recommended by the FSF producers) leads to an intake of MCs of 10 ng/kg bw/day for an adult of 60 kg. This value is more than four times lower than the tolerable daily intake of 40 ng/kg bw/day established by the WHO for chronic exposure. We can therefore conclude that the levels of contamination of spirulina produced by French small-scale producers are compatible with a daily consumption dose of 3 g with regard to the microcystin risk. However, the evolution of the levels of MCs observed between 2017 and 2021 should be taken seriously and efforts to monitor the MCs in spirulina products should be maintained.

Chemical methods, such as LC-MS/MS, measure only specific MCs using standards, and total MCs depending on the MC standard panel used and the composition of the MC structural congeners in the sample being analyzed. Adda-ELISA tests measure the total MC content—including all the MC congeners and nodularins—against a standard MC-LR. The Adda-ELISA method is also likely to react with degradation products of MCs, containing the ADDA group, and thus lead to overestimations of the MCs concentrations. This might overestimate the results from the toxicological point of view because of the reaction between antibodies and non-toxic derivates from MCs [45,46,47].

Nevertheless, several studies related to MC analyses on BGAS showed a good correlation between ELISA and LC-MS/MS methods (e.g., [48,49]). In our study, the comparison between MCs measured using ELISA and total congener MCs measured using LC-MS/MS shows large differences, the totals obtained via LC-MS/MS being between 2 and 18% of the MCs measured using ELISA. Furthermore, the levels of MC-LR measured in our study using LC-MS/MS are very low, and represent a small part of the total MCs. This last observation is in contradiction with the results obtained on klamath-based BGAS [14,22,48]. It is therefore likely that the samples of our study, which present high levels of MCs analyzed with the ELISA method, contain a significant proportion of congeners of the MCs not detected with the LC-MS/MS method or at least degradation products with the ADDA group. Future investigations should be conducted to identify these compounds.

ELISA, which is moderately expensive, quick to perform and capable of detecting a lot of MC structural congeners even unknown beforehand, is a good technique to determine the human exposure to MCs, and is therefore a relevant MC screening method [13]. The possible overestimates of the levels of MCs measured with this method increase food safety for the consumers. We therefore recommend that producers continue to use this method to monitor the levels of contamination of MCs in their production.

The taxonomic analysis of the cyanobacterial contaminants encountered in our study revealed 14 genera. In 2016, Vardaka et al. analyzed 31 samples of spirulina of global origin and could identify 15 OTUs of cyanobacterial contaminants by metagenetic analysis, with occurrence rates of 3 to 20% [33]. Only four of these OTUs correspond to the genera also found in our study—*Geitlerinema*, *Leptolyngbya*, *Phormidium* and *Pseudanabaena*—and conversely, eleven of these OTUs correspond to the taxa not present in our samples. In particular, the genus *Microcystis*, which is regularly associated with contamination by MCs, in others BGAS containing klamath (*Aphanizomenon flos aquae*) [14,22,25]. The absence of *Microcystis* in our samples is consistent with the difficulty for *M. aeruginosa* to develop under the spirulina culture conditions previously highlighted [50]. Nevertheless, according to the literature, eight of the cyanobacterial contaminant taxa identified in our study are potential producers of MCs: *Aphanocapsa* [51], *Cyanobium* [28,30], *Geitlerinema* [52,53,54], *Leptolyngbya* [54,55,56], *Merismopedia* [55], *Phormidium* [52,53,54], *Pseudanabaena* [52] and *Spirulina* [52]. This information must be interpreted with caution because the taxonomy of cyanobacteria is continuously evolving with the consequence of a possible confusion of the identification of the species studied in terms of toxin production.

In France, Belgium and Luxembourg, the cyanobacteria dominant taxa in natural environments are mainly *Plankthothrix*, *Microcystis*, *Anabaena*, *Aphanizomenon* and *Woronichinia* [57,58,59,60,61,62]. These major taxa were not identified in our study. Other taxa in our inventory, *Aphanocapsa*, *Merismopedia*, *Phormidium*, *Pseudanabaena* and *Romeria*, have sometimes been identified as the dominant taxon in the same territories [57,61]. It can be hypothesized that the spirulina cultures are partially protected from contamination by their extreme alkalinity and salinity conditions [40]. Conversely, the major contaminants encountered in spirulina cultures and products, *Cyanobium*, *Jaaginema* and *Planktolyngbya*, do not appear in the literature as being major cyanobacteria in these same territories. However, these major contaminants are present in practically all the farms and territories concerned. The inoculation of ponds by transfer of non-isolated cultures from one or more other farms is a common practice in the profession. This practice favors the spread of contaminants from farm to farm and could explain the wide geographical spread of the main contaminants. The diversity of other contaminants and levels of contamination (number of taxa) could be explained by contamination from the farm environment (maritime facade, surface water). It can also be assumed that farms can accumulate taxa through successive episodes of inoculation with a contaminated sample. We recommend that the profession uses cyanobacterial contaminant-free inoculums and avoids uncontrolled exchanges, in terms of contamination, between the farms.

The comparison of the populations of cyanobacterial contaminants of the spirulina culture samples and the harvested products shows that the latter are globally less contaminated and not representative of the contamination of the cultures. It is likely that the processing of the rehydrated dry samples is partly responsible for this poor representation and the observed absence of correlation between the levels of MCs and the populations of contaminants. Indeed, various steps of harvesting and transformation of the biomass, particularly including filtration, pressing, extrusion and drying, could alter the structure of certain cyanobacteria and make their identification difficult. It is therefore advisable to concentrate contamination monitoring efforts on culture media.

Harvesting methods also have an influence on the evolution of cyanobacterial populations between spirulina cultures and harvested products. Depending on the farms in our sample, biomass can be taken from the surface (skimming) or from the water column (pumping) and then filtered with filters that vary in operation (flat, vibrating or rotary filters) and mesh sizes (19 to 50 µm). These differences in the harvesting process are farm-specific, which is highlighted in the analysis of the evolution of *Cyanobium* populations before and after harvest. This finding suggests possible improvements in harvesting practices to reduce the load of contaminants, in particular picocyanobacteria, in the harvested biomass. The use of harvesting filters with continuous cleaning, such as rotary filters, and the rinsing of the biomass seem to be possible improvements.

In the absence of the correlation between the levels of MCs and the populations of cyanobacterial contaminants, this study could not identify the contaminant(s) responsible for the presence of the MCs. Genomic analysis will be necessary to identify those strains. Regarding spirulina, two consecutive studies by the same author claim to have found the MCs in an isolated culture of two strains of *Arthrospira fusiformis* [37,38]. The spirulina cultured in these studies came from lakes Sonachi and Bogoria (Kenya), in which there were also other microcystin-producing cyanobacteria, including picocyanobacteria (*Syncechococcus* sp., *Synechocystis* sp.). The methods described in those works do not specify the measures to ensure that the cultures were well isolated. Moreover, at the same time, these results were not confirmed on other ecotypes of *A. fusiformis* from the same region: lakes Simbi, Nakuru and Elmenteita [37,38,63]. Additionally, studies using molecular analysis methods have consistently demonstrated the absence of complete microcystin-related metabolic pathways in various strains of *Limnospira* (*Arthrospira*) sp. [63,64,65,66,67]. Finally, the low levels of MCs in the spirulina samples of this study compared to the levels of the analyses on cyanobacteria recognized as producing MCs (e.g., *Microcystis)* [68] seem to rule out the hypothesis of the presence of producing strains of MCs in the cultivar samples of our study.

The opportunity to analyze the data from a large number of samples has enabled us (1) to establish the situation of microcystin contaminations that can affect the production of French spirulina; (2) to obtain information on the origin of its contaminations and the methods used for their monitoring; (3) and to propose improvements to the French profession and, by extension, to the rest of the world. These recommendations concern biomass inoculation, harvesting practices, as well as the monitoring of cyanobacterial contamination and MC levels. We also highlighted the lack of knowledge on the microcystin variants produced by the cyanobacterial contaminants present in the spirulina cultures.

## 4. Materials and Methods

### 4.1. Data Collection

The data collected for this study comes from spirulina producers, members of the Fédération des Spiruliniers de France (FSF), who have agreed to share the anonymized results of their analyses.

Data from total cyanobacteria counts carried out in 2017, 2019, 2020 and 2021 on 437 dry spirulina (flakes) samples and 102 spirulina culture samples were used to establish the inventory of cyanobacterial contaminants present in 95 farms.

Seventy-five of these farms have been identified and located. Data from these farms, including 233 counts of cyanobacteria in spirulina products and 102 counts in spirulina culture samples, enabled us to establish the geographic distribution of the contaminants.

Cyanobacteria datasets include colony counts expressed as a percentage of total cyanobacteria (including *Limnospira*) present in the samples. Biovolume data, also expressed as a percentage of total cyanobacteria, are available for 232 analyses performed in 2019 and 6 in 2021 on spirulina products.

The results of the ELISA MCs analyses, carried out each year between 2013 and 2021 on a total of 623 samples of dry spirulina, make it possible to examine the levels of MCs in spirulina-based products from 109 farms.

In 2017, total cyanobacteria counts, as well as ELISA MCs analysis, were performed on 100 spirulina culture samples and 57 spirulina products from 16 farms. This makes it possible to compare the presence of cyanobacteria and MCs before and after harvest.

In 2021, five samples of dry spirulina showing high values of MCs according to an ELISA analysis, were then analyzed with UHPLC-MS/MS. The results are used to compare the two analytical methods.

The types and quantities of data per year, as well as the number of farms from which this data originated, are summarized in Table 3.

### 4.2. Microcystin Analyses

ELISA analyses of microcystins were performed by the laboratory Limnologie sarl (Rennes, France) using the following method. The ELISA tests by indirect competition were carried out with the ELISA Microcystins ADDA (US EPA Official Method 546) Abraxis kit (ref. 1520011)) and according to the protocol of Lawrence et al. [48].

UHPLC-MS/MS analyses on microcystins were performed by the public laboratory Labocea (Plouzane, France) using their protocol adapted from Turner et al. [69]. After cell lysis of the sample, the microcystins were analyzed on a UHPLC/MS-MS XEVO-TQS from Waters. The separation is carried out according to a gradient of mobile phases composed of water and acetonitrile. A calibration range is established for each molecule quantified in each series. Internal quality controls are placed every 10 samples. The acceptability criteria for the blanks, the range and quality controls are systematically checked. Internal standards allow for quantification. The retention times and m/z transition ratios are checked for each molecule.

### 4.3. Cyanobacteria Enumeration

The counts of cyanobacterial contaminants were performed by the laboratory Limnologie sarl (Rennes, France) using the following methods. The identifications of the genera of cyanobacteria were carried out with classical methods of morpho-taxonomy [70,71,72,73,74]. Cyanobacteria counting was performed using the Utermöhl method [75,76]. For non-colonial cyanobacteria (e.g., *Cyanobium*), each cell was counted as a colony. The enumerations are expressed as a percentage of colonies of each taxon compared to the total colonies of cyanobacteria (contaminants and *Limnospira*). The samples of dry spirulina, in the form of pieces of spaghetti (or flakes) approximately 1.5 mm in diameter, were rehydrated with demineralized water, before the microscopic observations.

### 4.4. Review of MCs in Spirulina Products

A search for publications related to the detection and quantification of microcystins in spirulina products was carried out in PubMed, ScienceDirect and Google Scholar databases, using the following query: (“BGA” OR “spirulina” OR “arthrospira” OR “limnospira”) AND (“microcystin” OR “cyanotoxin”). The articles or results that do not identify the nature of the samples as being composed exclusively of spirulina have been discarded.

### 4.5. Data Analysis

To calculate the averages of the levels of MCs, the results “below the limit of quantification (LQ)” (0.15 ppm with the ELISA method for dry spirulina, 0.15 ppb for water sample like spirulina culture) were set at the central value between zero and the LQ (i.e., 0.075 ppm for dry spirulina). The annual mean MC levels were compared with Tukey’s test. The correlations between the MC and contaminant levels were carried out with the methods of Pearson and Kendall. The comparisons of pairs of means were carried out with the Welch two-sample t-test.

Data analyses were performed using R 4.1.3 and RStudio 2023.03.0 with packages *car* version 3.0-13 and *multcomp* version 1.4-19. The figures were made using packages *ggplot2* version 3.3.6 [77] and *ggmap* version 3.0.0. [78], with maps provided by http://maps.stamen.com (accessed on 20 April 2023).

## Figures and Tables

**Table 1:** Microcystins ELISA assays versus UHPLC-MS/MS analysis of dry spirulina products.

	ELISA	UHPLC-MS/MS									
Farm ID	MC-LReq	MCs total ^1^	MC LR	dMC LR	MC LA	MC RR	dMC RR	MC LF	MC YR	MCLW	MC LY
F031	1.31	0.022	0.001	ND	ND	ND	ND	0.009	ND	0.012	ND
F055	1.25	0.127	0.008	0.012	ND	0.010	0.007	0.010	0.014	0.026	0.020
F075	1.22	0.227	ND	0.020	ND	ND	ND	0.007	ND	0.150	0.050
F031	1.20	0.170	0.015	0.018	0.016	0.005	0.025	0.025	0.007	0.048	0.020
F056	0.60	0.033	ND	ND	0.008	ND	0.009	0.009	0.004	0.011	ND

^1^ Total values of the nine MC variants analyzed. All values expressed in µg/g DW. ND: not detected.

**Table 2:** Detection of mycrocystins (MCs) in spirulina-based products.

Toxins	Method ^1^	Results	Market	References
MCs	ELISA	0.21 ppm ± 0.163 [<0.15–1.31] (361/623 samples)	France	This study
MCs	ELISA	0.15 ppm ± 0.08 [0.06–0.32] (8/8 samples)	USA	[14]
MCs	ELISA	0.52 ppm ± 0.77 [0.0–2.12] (6/7 samples)	USA	[14]
MCs	ELISA	0.047 ppm MCs [0.028–0.078] (3/3 samples)	Taiwan	[16]
MCs	ELISA	0.1 ppm (1/1 sample); < 0.01 ppm (0/4 samples)	Germany	[17]
MCs	PPIA	<0.25 ppm (0/4 samples)	USA	[18]
MCs	UHPLC-MS/MS	[0.022–0.227 ppm] (5/5 samples). Detailed in Table 1.	France	This study
MC-LR, MC-RR	MALDI-TOF	Not detected (0/12 samples)	Australia	[19]
MC-LR, MC-RR, MC-YR	LC-MS/MS	0.014 ppm ± 0.027 MC [0.002–0.163] (34/34 samples)	China	[20]
MC-LR, RR, YR, LA	UHPLC-TOF	Not detected (0/10 samples)LD: 0.1 ppm (0.2 ppm for MC-RR)	Switzerland	[21]
MC-LR, RR, YR, LW, LF, LA	LC-MS/MS	Not detected (0/5 samples)(LD 0.01–0.20 ppm depending on MC congener)	Germany	[17]
MC-LR, RR, YR, LW, LF, LA, LY	LC-MS/MS	Not detected (0/6 samples). 1.2 ppb < LQ < 15 ppb depending on MC variants	Italia	[22]
MC-LR, RR, FR, LY	LC-HRMS	MCs detected in 6 samples (6/17). No quantification available	India	[23]
MC-LR, RR, YR, LW, LF, LA, LY	LDTD-APCI-HRMS	0.25, 0.6 and 2.5 ppm total MCs DW; (3/11).	USA	[24]
MC-LR, RR, LA, LF, LY, LW, YR	UHPLC-MS/MS	Not detected (0/19 samples). LD 22.5 ppb	Belgium	[25]
MC-LR, MC-RR	HILIC-MS/MS	<0.015 ppm (0/6 samples)	Spain	[26]

^1^ ELISA: enzyme-linked immunosorbent assay; LDTD: laser diode thermal desorption; APCI: atmospheric pressure chemical ionization; HRMS: high-resolution mass spectrometry; MS: mass spectrometry, UHPLC: ultra-high performance liquid chromatography; HILIC: hydrophilic interaction liquid chromatography; PPIA: protein phosphatase inhibition assay; LD: limit of detection. Mean ± SD [min.–max. values]. Positive samples/total sample number.

**Table 3:** Sample collection from spirulina producers: results of the analyses of microcystins (MCs) through ELISA and UHPLC-MS/MS methods, and the enumeration of cyanobacteria of samples of dry spirulina (Dry S.) and spirulina cultures (S. Culture); number of farms having provided their results. The number between the brackets refers to data including location information.

Year	MCs Analyses				Cyanobacteria Enumeration		
	ELISA			UHPLC			
	Dry S.	S. Culture	Farms	Dry S.	Dry S.	S. Culture	Farms
2013	13		13				
2014	5		5				
2015	9		9				
2016	8		8				
2017	57 (57)	105 (105)	16 (16)		57 (57)	100 (100)	16 (16)
2018	153 (153)		38 (38)				
2019	233 (30)		69 (30)		232 (30)		69 (30)
2020	50 (48)		27 (26)		49 (47)		26 (25)
2021	95 (95)		40 (40)	5 (5)	99 (99)	2 (2)	41 (41)
All	623 (383)	105 (105)	109 (89)	5	437 (233)	102 (102)	95 (75)

## Data Availability

Not applicable.
